# Design and evaluation of a rodent-specific focal transcranial magnetic stimulation coil with the custom shielding application in rats

**DOI:** 10.3389/fnins.2023.1129590

**Published:** 2023-04-17

**Authors:** Li Liu, Ming Ding, Junfa Wu, Yuwen Zhang, Shaoqian Guo, Nianhong Wang, He Wang, Kewei Yu, Yuanfeng Weng, Lu Luo, Jingjun Zhang, Quan Zhang, Kai Qiu, Yi Wu, Xiao Xiao, Qun Zhang

**Affiliations:** ^1^Department of Rehabilitation Medicine, Huashan Hospital, Fudan University, Shanghai, China; ^2^Behavioral and Cognitive Neuroscience Center, Institute of Science and Technology for Brain-Inspired Intelligence, Fudan University, Shanghai, China; ^3^Institute of Science and Technology for Brain-Inspired Intelligence, Fudan University, Shanghai, China; ^4^Nanjing Vishee Medical Technology Co., Ltd., Nanjing, China

**Keywords:** rTMS, high magnetic permeability material, electromagnetic shielding, finite element method, fluorescence imaging, fMRI

## Abstract

Repetitive TMS has been used as an alternative treatment for various neurological disorders. However, most TMS mechanism studies in rodents have been based on the whole brain stimulation, the lack of rodent-specific focal TMS coils restricts the proper translation of human TMS protocols to animal models. In this study, we designed a new shielding device, which was made of high magnetic permeability material, to enhance the spatial focus of animal-use TMS coils. With the finite element method, we analyzed the electromagnetic field of the coil with and without the shielding device. Furthermore, to assess the shielding effect in rodents, we compared the c-fos expression, the ALFF and ReHo values in different groups following a 15 min 5 Hz rTMS paradigm. We found that a smaller focality with an identical core stimulation intensity was achieved in the shielding device. The 1 T magnetic field was reduced from 19.1 mm to 13 mm in diameter, and 7.5 to 5.6 mm in depth. However, the core magnetic field over 1.5 T was almost the same. Meanwhile, the area of electric field was reduced from 4.68 cm^2^ to 4.19 cm^2^, and 3.8 mm to 2.6 mm in depth. Similar to this biomimetic data, the c-fos expression, the ALFF and ReHo values showed more limited cortex activation with the use of the shielding device. However, compared to the rTMS group without the shielding application, more subcortical regions, like the striatum (CPu), the hippocampus, the thalamus, and the hypothalamus were also activated in the shielding group. This indicated that more deep stimulation may be achieved by the shielding device. Generally, compared with the commercial rodents’ TMS coil (15 mm in diameter), TMS coils with the shielding device achieved a better focality (~6 mm in diameter) by reducing at least 30% of the magnetic and electric field. This shielding device may provide a useful tool for further TMS studies in rodents, especially for more specific brain area stimulation.

## Introduction

Transcranial magnetic stimulation (TMS) is a non-invasive procedure that uses a magnetic field to modulate neuronal activity (Salinas et al., 2016; Toledo et al., 2021). Repetitive TMS (rTMS) has been used as an alternative treatment for various neurological disorders, usually when other treatments are ineffective (Lefaucheur et al., 2020). Although, the molecular mechanisms underlying TMS-induced neurorecovery have been systematically studied in rodent models (Xing et al., 2022), most TMS studies in rodents were based on the whole brain stimulation with the commercial coils (Roth et al., 2007; Vahabzadeh-Hagh et al., 2012; Guerra et al., 2020). The lack of rodent-specific focal TMS coils restricts the proper translation of human TMS protocols to animal models. However, neither intensity reduction nor miniature coil construction to increase the coil focality is perfect, because low-intensity is not sufficient to mimic the human stimulation conditions, while the miniature coil could not bear long-term stimulation (Cohen and Cuffin, 1991; Rodger et al., 2012; Tang et al., 2016). Therefore, given the three important TMS parameters, i.e., pulse capacitor(C), high-voltage power (U) and inductance coil (Ls) are mutually interacted, it is not easy to make a TMS coil that is localized and with a high-magnetic and high-electric field.

Previous studies have reported that shielding device with high permeability material (silicon) may be useful to increase the coil focality approximately 50% without changing the coil type (Boonzaier et al., 2020). However, the above shielding material was not able to withstand prolonged TMS stimulation due to overheating. Accumulating evidence suggests that “thin, light, wide and strong” absorbing materials were more suitable for the TMS electromagnetic shielding (Kim et al., 2006). Meanwhile, other studies have shown that using highly permeable soft magnetic ferrite could improve focalization of the coil, whereas they only simulated the distribution of the electric field, and no *in vivo* data have been available to reveal the real neuron activation in TMS stimulation with or without the shielding device (Zhang et al., 2013; Zhao et al., 2015). As there is large discrepancies between *in vivo* and bionic simulation data, more work needs to be done to reveal the actual changes in the brain.

Therefore, in this study, in addition to the magnetic and electric field distribution analysis with the Finite element method (FEM), we further used the immunofluorescence (IF) and functional magnetic resonance imaging (fMRI) to assess the aftereffects of a 15 min 5 Hz rTMS paradigm in rats with or without the shielding device.

## Materials and methods

### The shielding device

**Figure 1:**
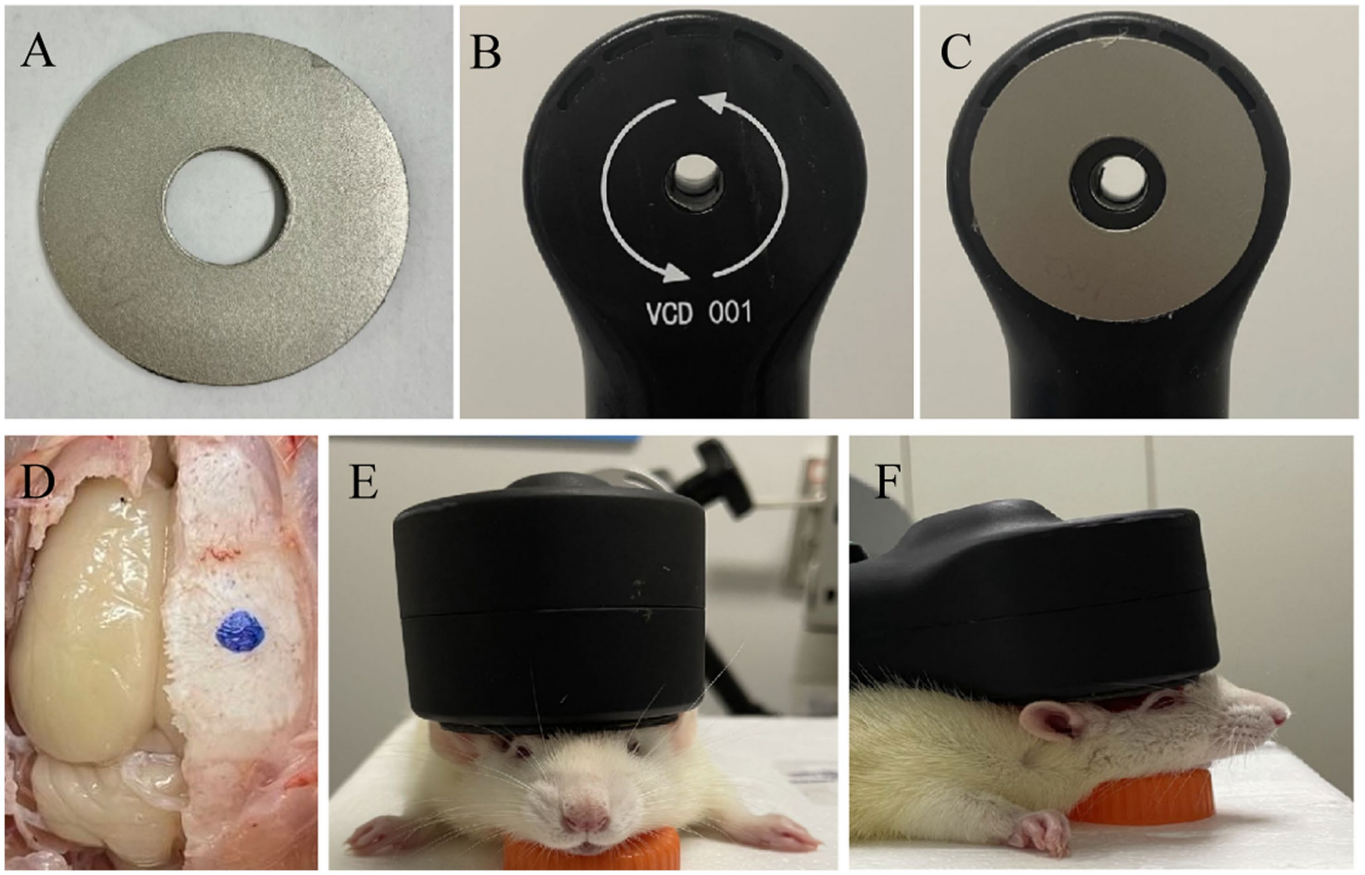
Schematic of TMS stimulation and shielding material. **(A)** Shielding device. **(B)** TMS coil. **(C)** The TMS coil with high magnetic permeability material. **(D)** The center of TMS stimulation at the blue dot. **(E,F)** Images of TMS stimulation on anesthetic rats.

Absorbing material, a sort of electromagnetic (EM) shielding material, is thin coating with light weight. It has a strong absorbing performance (Wen et al., 2014; Huang et al., 2021; Nuhiji et al., 2021; Du, 2022). Through magnetic loss, dielectric loss, or resistive loss, the absorbing material can reduce the EM field as required (Zhang et al., 2020). The magnetic loss material (the ferrite, magnetic metal, alloys, etc.) and the conductive loss material (the carbon material, graphene, MXene, *SiC*, etc.) can convert the electromagnetic force into heat directly (Gao et al., 2020), while the dielectric loss of material, like the TiO2, MnO2, etc. will consume the electromagnetic and further convert it into heat (Qin et al., 2022). In this study, we used a composite absorbing material made by mixing magnetic powder and epoxy resin to make the custom-made shielding device. Furthermore, a 15 mm diameter hole was designed in the center of the shielding device to realize the focal stimulation. Specifically, the shielding device is circle with a 45 mm outer diameter and a 2 mm thickness (Figures 1A,B). The TMS coil center was tangentially attached above the right brain with the shielding device pasted on the back (Figures 1C–F).

### Animals

Female Sprague–Dawley rats of clean grade (230–250 g) from Shanghai JSJ Company were used in this study. All the animals were housed in an environment with a temperature of 22–25°C, relative humidity of 65 ± 5%, and a light/dark cycle of 12/12 h and had free access to food and water. All animal studies (including the rats euthanasia procedure) were reviewed and approved by Fudan University Animal Welfare and Ethics committee (Ethical permit numbers: 2020 Huashan hospital JS-151).

### rTMS treatment

**Figure 2:**
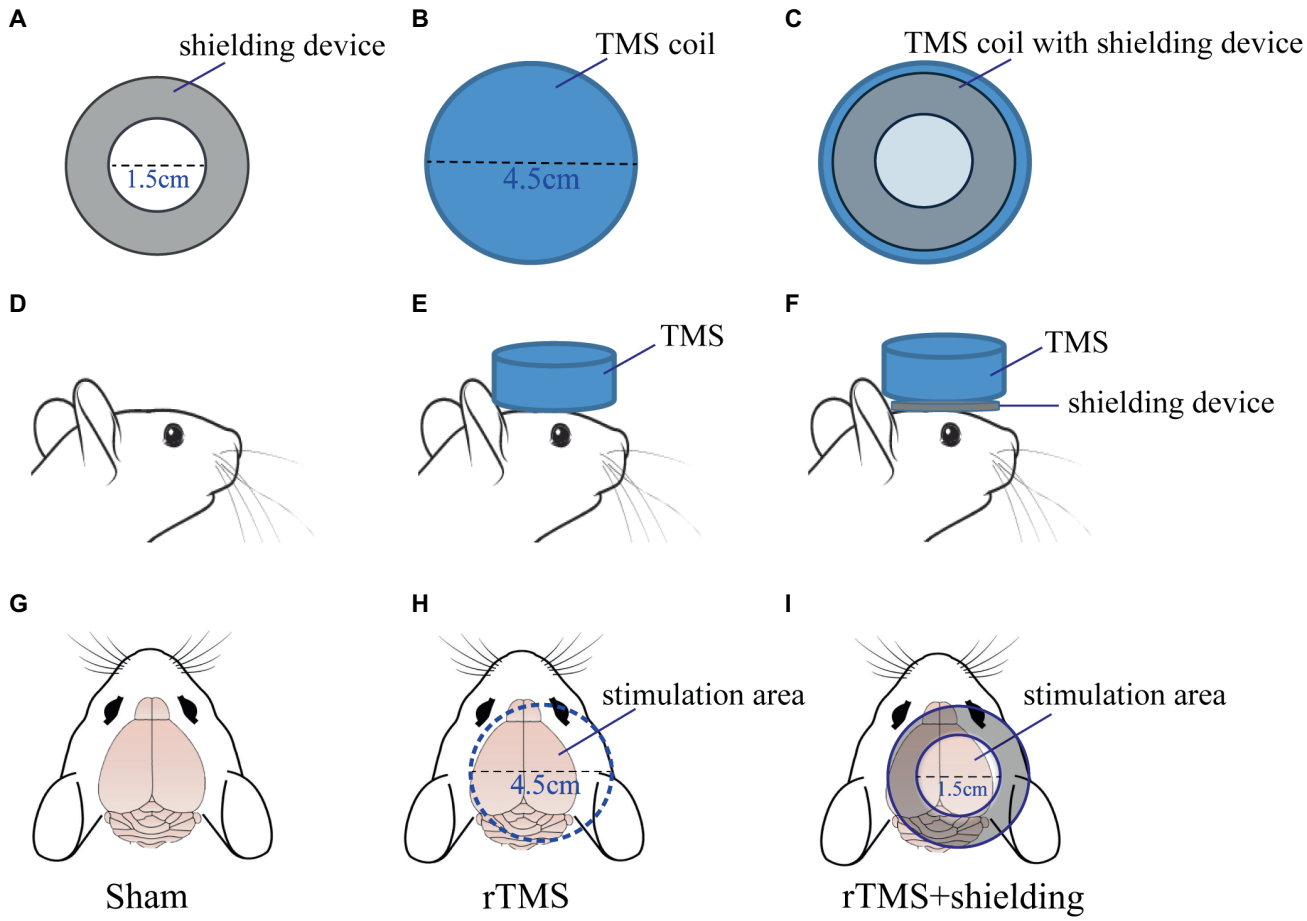
The TMS coil and shielding device. **(A)** The shielding device. **(B)** The TMS circular coil. **(C)** The TMS coil with the shielding device. **(D,G)** The sham group. **(E,H)** the rTMS group. **(F,I)** The rTMS +shielding group.

The rats were randomly divided into three groups: the rTMS with the custom shielding group (rTMS+shileding), the rTMS with a plastic board group (rTMS), and the sham stimulation group (Sham). Before the rTMS stimulation, the rats were anesthetized with 1% isoflurane in oxygen air and then fixed in the stereotaxic apparatus. The magnetic stimulator (VISHEE-TMS-013, Nanjing VISHEE Medical Technology, Nanjing, China) with a circular coil (inner diameter: 15 mm, outer diameter: 45 mm) was used to deliver the rTMS. In this study, the coil material was the 38-turns oxygen-free copper used in many studies which generates a vertical electric field. When TMS stimulation was performed, the TMS coil was fixed to the stereotaxic apparatus, and the center of the coil was attached tangentially to the rat’s right lateral parietal association (LPtA) cortex (coordinates: ~3 mm lateral to the midline and ~ 3.36 mm caudal to Bregma). In addition, the center of the coil was hollowed, we could accurately locate the target coordinates to avoid displacement. For the rTMS+shileding group, the coil was attached with the custom shielding device which the inner radius was 7.5 mm. Therefore about 4.5 mm of the left hemisphere was exposed under the device hole. Meanwhile in the rTMS group, the coil was attached with a plastic board of the same weight. For the sham group, the rats were administered with an identical manipulation without real TMS stimulation (instead, they received an auditory stimulus) (Figure 2). In our study, 5 Hz rTMS protocol consisted of stimulation for 2 s followed by rest for 13 s and was repeated 60 times, at 35% maximum stimulator output of 15 min (600 pulses).

### Immunofluorescence staining

Approximately 1.5 h after the rTMS paradigm, the rats were perfused with 0.9% sodium chloride followed by 4% paraformaldehyde. The brain tissues were fixed with 4% paraformaldehyde for 12 h before transferring into a 30% sucrose solution. Coronal sections were cut on a freezing microtome. The sections (30 μm) were washed with PBS three times, followed by 0.3% Triton X-100 incubation for 10 min and 1% BSA for 1 h at room temperature. Samples were then incubated with rabbit anti-c-fos antibody (1:1000, 226003, synaptic system) at 4°C overnight and then with donkey anti-rabbit IgG H&L (Alexa Fluor® 594) (1:1000, Abcam, USA) for 1 h at room temperature. A confocal laser-scanning microscope (Olympus, FV3000) was used to assess the c-fos expression in different groups.

### MRI assessment

All the rats were further anesthetized with 1% isoflurane in oxygen air. The breathing and heart rate were monitored. The body temperature was kept by a water circulation system set at 37°C. *In vivo* whole-brain MRI images were acquired immediately after the rTMS (*n* = 5 per group) with an 11.7 T MRI scanner (Bruker, Ettlingen, Germany). A 4-channel surface array coil (Bruker BioSpin, Billerica, MA) was adopted to receive the magnetic resonance signals. The resting state functional MRI (rsfMRI) was acquired with a spin-echo echo-planer (SE-EPI) sequence: repetition time (TR) = 2000 ms, echo time (TE) = 12.8 ms, the field of view (FOV) =30 × 30 mm, and slice thickness = 0.5 mm. The anatomical image (T2 image) was acquired by a spin echo (Turbo-RARE) sequence. The T2 image sequence parameters were: TR = 5,000 ms, TE = 25 ms, FOV = 30 × 30 mm, and slice thickness = 0.5 mm.

### MRI data analysis

The MRI data were analyzed by the Statistical Parametric Mapping software (SPM12, University College London, U.K.), FMRIB Software Library (FSL), ANTs, and DPABI (a toolbox for Data Processing & Analysis for Brain Imaging). All the raw images were enlarged by a factor of ten to correlate the image dimensions to human images by SPM12. The rat brain mask was obtained by the ITK-SNAP (a toolbox for Data Processing & Analysis for Brain Imaging) and FSL. The slice timing, realignment, and normalization were processed by the *in vivo* functional template (SIGMA) using ANTs. After normalization, all images were smoothed using a Gaussian Kernel of 4 mm (FWHM).

The amplitude of low-frequency fluctuation (ALFF) and regional homogeneity (ReHo) was calculated for the traditional low-frequency band (0.01–0.08 Hz) by DPABI. One-way ANOVA (two-tailed) multiple comparisons test was used to analyze the ALFF and ReHo values among the sham group, the rTMS group, and the rTMS+shielding group. The resulting statistical map was set at *p* < 0.05 (with correct).

### Statistical analysis

GraphPad Prism 8 and Image-J software were used to analyze the c-fos staining data. Data were presented as mean ± SEM using unpaired two-tailed Student’s *t*-test. A value of *p* <0.05 was considered significantly different.

## Results

### The shielding device increases the TMS coil focality

**Figure 3:**
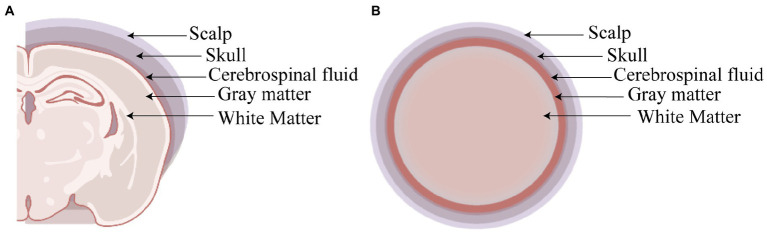
The rat head model. **(A)** Coronal section of rat head model. **(B)** The sphere shape of head model.

**Figure 4:**
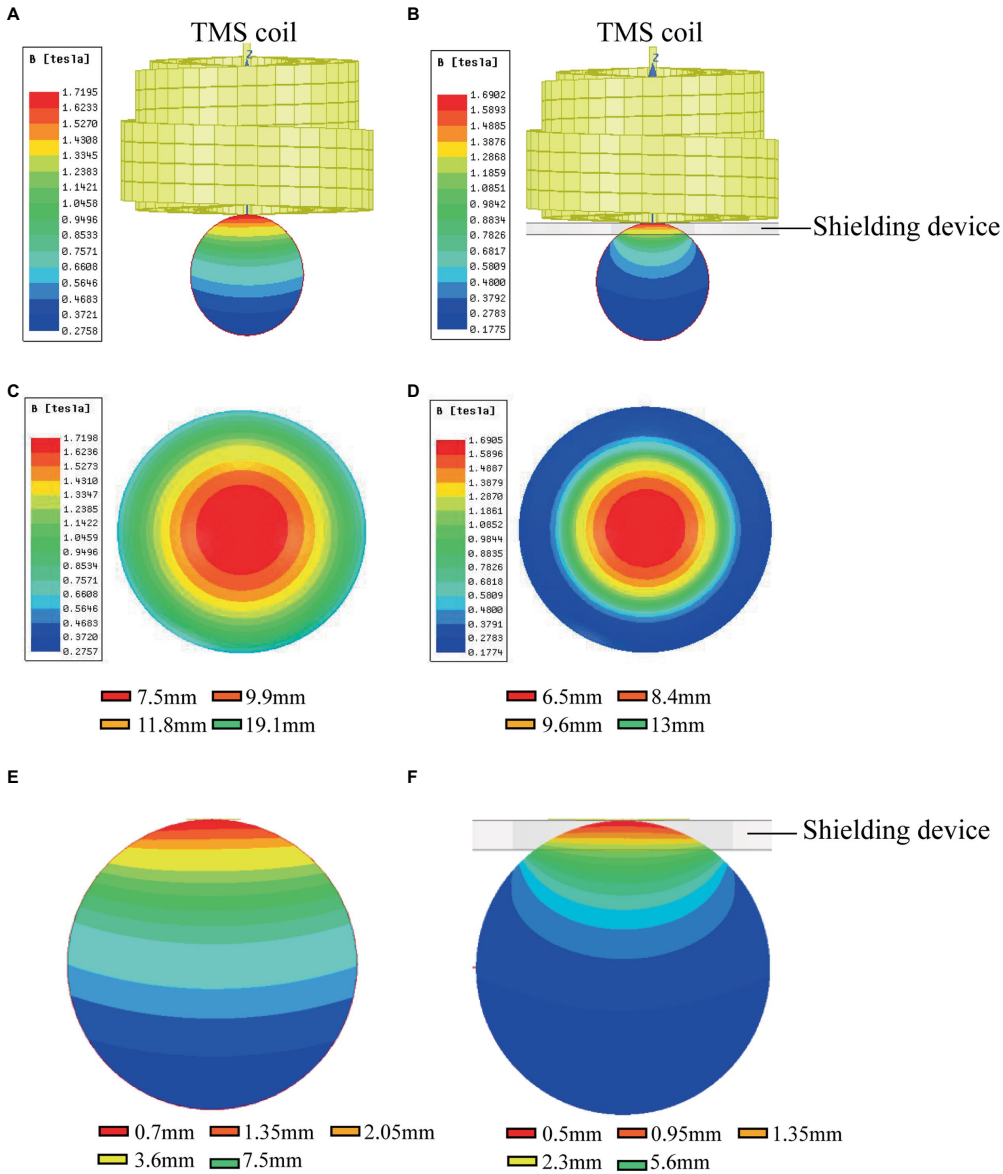
The magnetic fields of TMS coil with or without shielding device. **(A)** The sketch of the TMS coil without shielding device. **(B)** The sketch of the TMS coil with shielding device. **(C)** An overhead view of the magnetic field without the shielding device. **(D)** An overhead view of the magnetic field with the shielding device. **(E)** A coronal view of the magnetic field without the shielding device. **(F)** A coronal view of the magnetic field with the shielding device.

**Figure 5:**
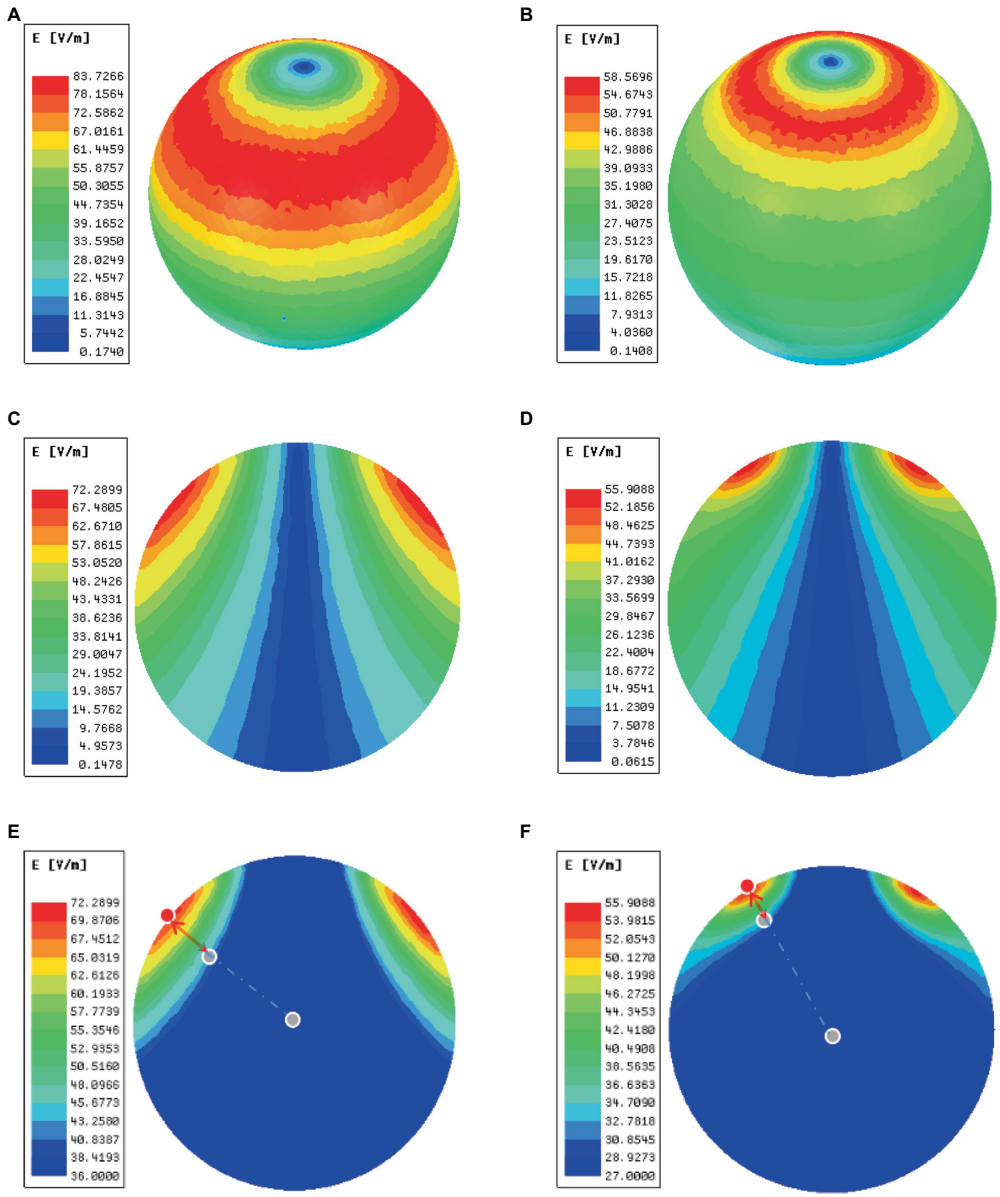
The electric fields of TMS coil with or without the shielding device. **(A)** An overhead view of the electric field without the shielding device. **(B)** An overhead view of the electric field with the shielding device. **(C)** A coronal view of the electric field without the shielding device. **(D)** A coronal view of the electric field with the shielding device. **(E)** The area (green area) and depth (red arrow) of electric field distribution without the shielding device. **(F)** The area (read area) and depth (red arrow) of electric field distribution with the shielding device. The red dots represent the electric field (*E* = *E*_max) of the cortical surface, and the gray dots in the center of the sphere represents the electric field (*E* = *E*_max/2) of the brain.

In order to validate the high magnetic permeability material shielding effect, we analyzed the electromagnetic field of the coil by the finite element method (FEM) software ANSYS. As shown in Figures 3A,B, a concentric sphere model was established to mimic the rat head model. The model consisted of 5 parts, including the scalp, the skull, the cerebrospinal fluid (CSF), the gray matter (GM), and the white matter (WM). The specific parameters are shown in Table 1, including the radius, the conductivity, and the relative permittivity. With the shielding device, the TMS magnetic field was more focused. As shown in Figures 4A,B, the whole magnetic field distribution was smaller than the non-shielding group. Figures 4C,D shown that the 1 T magnetic field (green area), which is small enough to induce the neuron activity (Boyer et al., 2022), is 19.1 mm vs. 13 mm in diameter (rTMS vs. rTMS+shielding), while the magnetic field over 1.5 T (red area) is 7.5 mm vs. 6.5 mm (rTMS vs. rTMS+shielding). Furthermore, compared with the rTMS group, the depth of 1 T magnetic field (green area) was reduced from 7.5 to 5.6 mm, while the magnetic field over 1.5 T (red area) was reduced from 0.7 to 0.5 mm with the shielding device (Figures 4E,F). The electric field distributions of TMS coil with or without shielding device were significant differences, the results shown better focal stimulation in coil with shielding. Compare with the rTMS group, the area of electric field (read area) was reduced from 4.68cm^2^ to 4.19cm^2^, while the maximum *E* values in the center was reduced 30%, from 83 V/m to 58 V/m with the shielding device (Figures 5A–D). In addition, the depth (red arrow) of electric field was reduced from 3.8 mm to 2.6 mm, as well as the volume reduced from 1.78 cm^3^ to 1.09 cm^3^ (rTMS vs. rTMS+shielding; Figures 5E,F).The results indicated that a smaller focality with an identical core stimulation intensity was achieved in the shielding device. However, we should note that a different magnetic and electric field distribution occurred under the shielding material. A more vertical magnetic and electric field was produced by the device.

**Table 1:** Tissue permittivity and conductivity.

Tissue name	Radius (mm)	Conductivity	Relative permittivity
Scalp	10	0.31061	25,809
Skull	9.7	0.02038	30,382
CSF	8.9	2	109
GM	8.8	0.10696	26,640
WM	7.4	0.65578	57,359

CSF, Cerebrospinal Fluid GM, Gray Matter WM, White Matter.

### The shielding device reduces the rTMS-induced c-fos activation in the RSD, PtA, and S1 cortex

**Figure 6:**
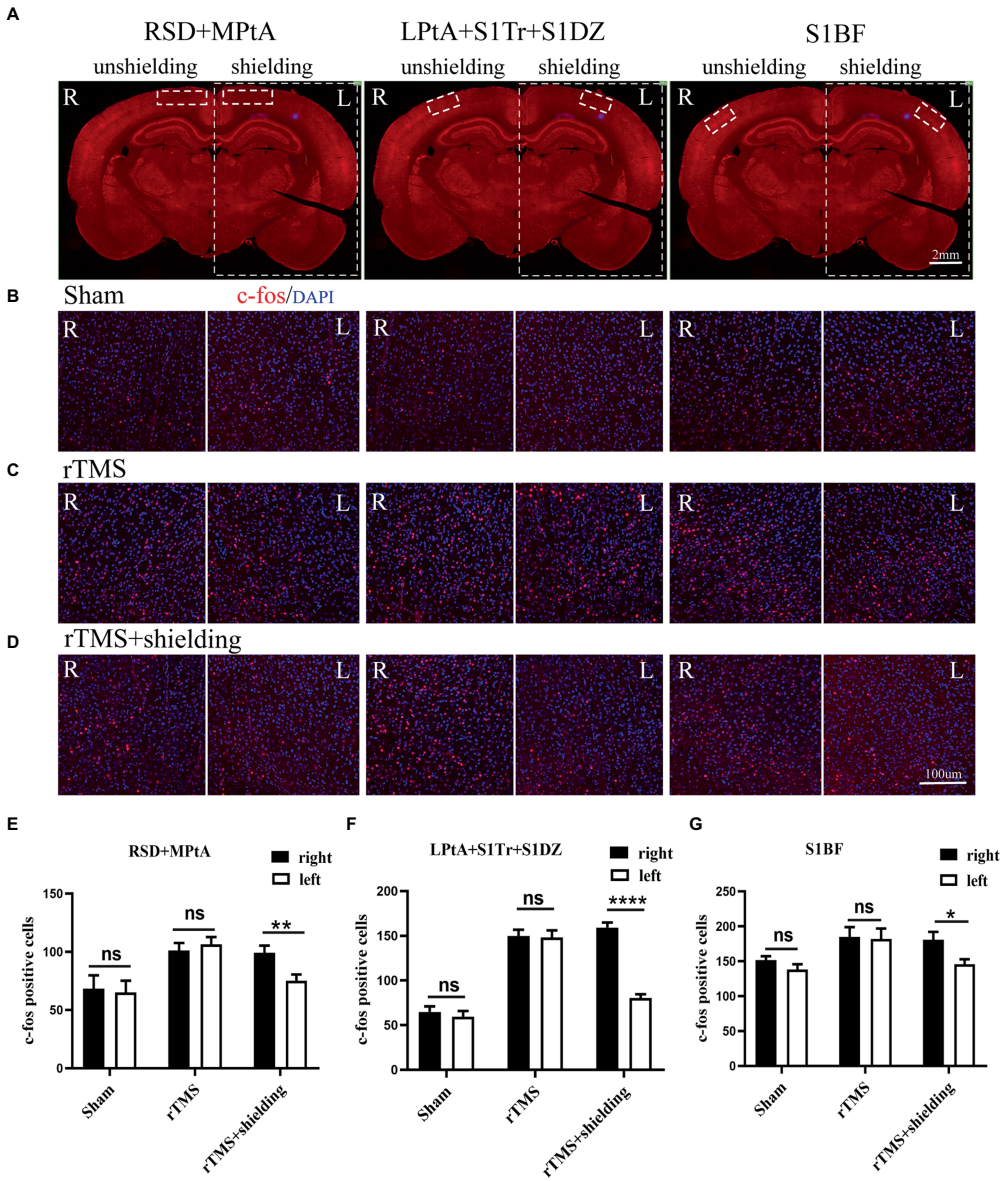
The c-fos expression in the cortex. **(A)** A schematic illustration of three different regions in the left and right cortex on a brain slice; the left cortex is under the shielding material, while the right cortex is exposed to the magnetic field. The scale bar is 2 mm. **(B–D)** Representative fluorescent images of three different regions in the left and right cortex of the sham group **(B)**, the rTMS group **(C)** and the rTMS+shielding group **(D)**. The scale bar is 100 μm. **(E–G)** Quantitative analyses of the c-fos expression in each group. Data are shown as the mean ± SEM (*n* = 3). *p < 0.05, **p < 0.01, ****p < 0.0001. RSD: retrosplenial dysgranular cortex; MPtA: medial parietal association cortex; LPtA:lateral parietal association cortex; S1Tr: primary somatosensory cortex, trunk region; S1DZ: primary somatosensory cortex, dysgranular region; S1BF: primary somatosensory cortex, barrel field.

**Figure 7:**
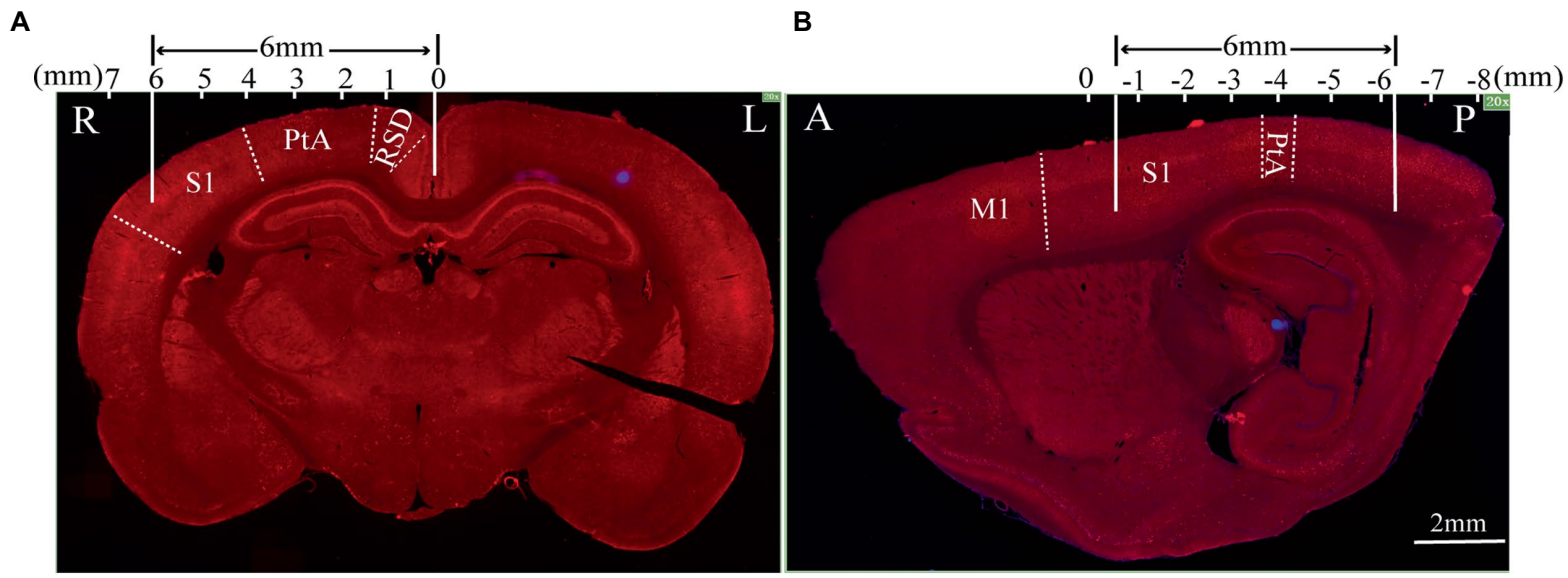
The effective stimulation area of the TMS coil with the shielding device. **(A)** Coronal section; **(B)** sagittal section.The scale bar is 2 mm. S1: primary somatosensory cortex; PtA: parietal association cortex; RSD: retrosplenial dysgranular cortex; M1: primary motor cortex.

To further test the neuronal activity effect of TMS with shielding device *in vivo*, we assessed the c-fos expression, an indicator of neuronal activity that peaks 1–3 h post-stimulus exposure (Olsen et al., 2022). Compared with the sham group (Figure 6B), 1.5 h after one session (600 pulse) of rTMS increased the relative c-fos expression in the rTMS group (Figure 6C) in three regions (Figure 6A), i.e., the right RSD + MPtA (Sham vs. rTMS, *p* = 0.018), the right LPtA+S1Tr + S1DZ (Sham vs. rTMS, *p* < 0.0001) and the right S1BF (Sham vs. rTMS, *p* = 0.026). Moreover, the left RSD + MPtA, LPtA+S1Tr + S1DZ and S1BF also showed higher expression than the sham group. (Sham vs. rTMS, value of *p* is *p* = 0.004, *p* < 0.0001, *p* = 0.023, respectively). Since the TMS coil was attached to the right hemisphere, in the rTMS+shielding group, the right cortex (unshielded region) was found to have a higher c-fos expression compared to the left cortex (shielded region), including the RSD + MPtA (right vs. left, *p* = 0.009), the LPtA+S1Tr + S1DZ (right vs. left, *p* < 0.0001) and the S1BF (right vs. left, *p* = 0.011; Figure 6D), while no difference was found between the two hemispheres in the sham group and rTMS group (*p* > 0.05; Figures 6E–G). The c-fos was the most expressed in the right RSD, PtA and S1 on the coronal section, and S1 and PtA on the sagittal section, while a low c-fos expression was detected around the area. This indicated that the effective stimulation area was approximately 6 mm in diameter with a shielding material (Figures 7A,B).

### The shielded region shows less aftereffects following rTMS in brain structure and function

**Figure 8:**
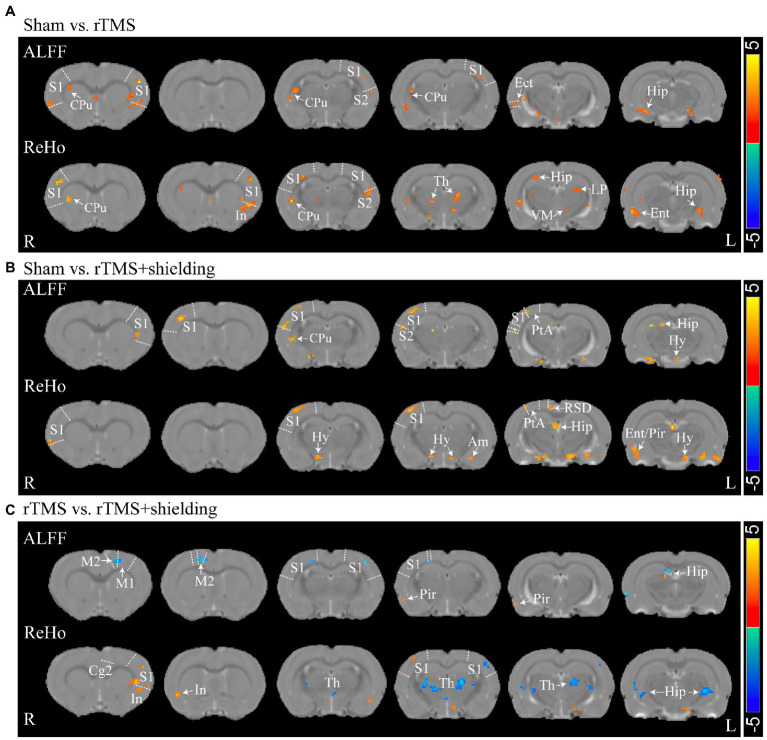
Statistical maps of a voxel of ALFF and ReHo analysis. **(A)** The ALFF and ReHo analysis between Sham (*n* = 5) and rTMS groups (*n* = 5). **(B)** Comparison of the ALFF and ReHo analysis between Sham and rTMS+shielding groups (*n* = 5). **(C)** Comparison the ALFF and ReHo analysis between rTMS and rTMS+shielding groups. S1: primary somatosensory cortex; S2: secondary somatosensory cortex; CPu: Striatum; Th: thalamus; Hy: hypothalamus; Am: amygdaloid; Ect: ectorhinal cortex; LP: lateral posterior thalamic nucleus; PtA: parietal association cortex; Hip: hippocampus; RSD: retrosplenial dysgranular cortex; M2: secondary motor cortex; Ent: entorhinal cortex; Pir: piriform cortex; VM: ventromedial thalamic nucleus. In: insular; Cg2: cingulate cortex, area 2.

For assessment of the aftereffect of rTMS stimulation, fMRI was used to acquire high-spatial-resolution data. We measured the rsfMRI signals among the sham group, rTMS group, and rTMS+shielding groups. Compared to the sham group, the ALFF and ReHo values of the rTMS group showed that both the left and right hemispheres were activated by the rTMS, including the primary somatosensory cortex (S1), the secondary somatosensory cortex (S2), the striatum (CPu), the insular, the hippocampus, the thalamus and the hypothalamus (Figure 8A). However, compared to the sham group, the ALFF and ReHo values were significantly higher in the right cerebral hemisphere in the rTMS+shielding group. Different from the rTMS group, more right hemispherical area showed higher values in the rTMS+shielding group, like the retrosplenial dysgranular cortex (RSD) and parietal association cortex (PtA; Figure 8B). In addition, in comparison to the rTMS group, the ALFF and ReHo values in the right hemispheres were higher in the shielding group, including the piriform cortex (Pir) and S1, while in the left hemisphere, the values were obviously lower, like the S1 (Figure 8C).

## Discussion

In the present study, we have developed a new TMS shielding device capable of enhancing the spatial focus of the TMS circular coils for rodents use. The new application was verified in the rat cortex, including the RSD, PtA, and S1. The electromagnetic field distribution and the neuronal activation results indicated a significant decrease in rTMS stimulation area in the anesthesia rats with the shielding device. In view of these findings, we provided a useful tool for further accurate TMS studies in rodents.

Compared with the previous shielding materials like silicon, we used a much thinner, lighter, and stronger absorbing material to shield the electromagnetic of the TMS coil. The material has better adhesion and anti-corrosion with low eddy current and good thermal conductivity. The material produces little heat after long-term TMS. Moreover, the material is flexible, which allows us to customize the shape to meet the needs of different stimulation paradigm. Furthermore, although previous studies have reported the highly permeable soft magnetic ferrite could improve the figure-eight coil focalization, the electric fields distribution of TMS coil has not been further validated on humans or animals (Zhang et al., 2013; Zhao et al., 2015). In our study, besides the magnetic and electric field distribution with the FEM, we also further validated the shielding effect *in vivo* with the fMRI and c-fos staining.

The inner diameter of the hole in the shielding device was 15 mm, the effective stimulation area was only approximately ~6 mm in diameter. Compared with the commercial rodents’ TMS coil (15 mm in diameter), TMS coils with shielding devices achieved a better focality (~6 mm in diameter). With the center of the TMS coil aligned with the following coordinates: ~3 mm lateral to the midline and ~ 3.36 mm caudal to Bregma, only the RSD, PtA, and S1 were activated. This indicated that more specific brain area stimulation may be achieved by the shielding device, especially for those diseases involving the above brain areas, like stroke or other neurodegenerative diseases. Although the previous study reported a stimulation with 1 mm diameter (Meng et al., 2022), the electromagnetic field may not be enough to provide continuous stimulation because of heating. As shown in Figures 4C–D, the 1 T magnetic field, which is enough to induce the neuron activity, was 19.1 mm vs. 13 mm in diameter (rTMS vs. rTMS+shielding), thus indicating a lower focality that was achieved with our shielding device. Moreover, without excessively changing the core stimulation, the magnetic field over 1.5 T (red area) was just 7.5 mm vs. 6.5 mm (rTMS vs. rTMS+shielding).Similarly, the area of electric field was reduced from 4.68cm^2^ to 4.19cm^2^, and the depth reduced from 3.8 mm to 2.6 mm (rTMS vs. rTMS+shielding). However, we should note that a different magnetic and electric field distribution occurred under the shielding material with a more vertical magnetic and electric field into the brain tissue. This may well explain the fMRI data showing more hemispherical region activation in the shielding group, such as S1.

Furthermore, different from the c-fos results, we also observed multiple neuron activation in the subcortical regions and S2 besides the cortical activation, like the striatum (CPu), the hippocampus, the thalamus, and the hypothalamus, which may be due to brain interconnection activation *via* transsynaptic way (Eldaief et al., 2011; Halko et al., 2014; Chen et al., 2020) since the primary somatosensory cortex (S1) sends a massive, topographically organized projection directly to the S2, striatum (CPu; Hur and Zaborszky, 2005; Aronoff et al., 2010; Sun et al., 2021; Whilden et al., 2021), while parietal association cortex (PtA) and retrosplenial dysgranular cortex (RSD) are also connected with the thalamus and hypothalamus (van Groen and Wyss, 1992). The activation of CPu and the deep nuclei of the thalamus and hypothalamus may be due to the TMS stimulation in the S1 and other cortex.

The present study has several limitations. First, the ratio of the magnetic powder and epoxy resin, the TMS intensity and the frequency, the brain dielectric constants are all important variables that may affect the shielding effect, different models and TMS parameters may need accordingly adjustments. Secondly, we only explored the shielding effect by analyzing the neuronal activity and the BOLD response signals. However, motor evoked potential (MEP), a widely used tool to assess corticospinal conduction (Bestmann and Krakauer, 2015; Jiang et al., 2021; Wilson et al., 2021; Meng et al., 2022), may be another way to assess the shielding effect. Although the shielding was applied, we could locate more accurate M1 hotpots; yet, as noted above, attention should be paid to the effective neuron activity in the human head with our material parameters. Different kinds of head models may probably need a different but specific shielding material.

In summary, this study reported a novel TMS shielding device. Compared to the conventional animal TMS coil, this new tool provides a more focal and effective electric field induced in the rat hemisphere and may be used for future human translational TMS studies.

## Data availability statement

The datasets presented in this article are not readily available because the original contributions presented in this study are included in the article/supplementary material, further inquiries can be directed to the corresponding author. Requests to access the datasets should be directed to QunZ, zqun_888@163.com.

## Ethics statement

The animal study was reviewed and approved by Fudan University Animal Welfare and Ethics committee (Ethical permit numbers: 2020 Huashan hospital JS-151).

## Author contributions

LiL, YZ, YFW, LuL, and JZ performed experiments. MD analyzed the fMRI data. SG, QuanZ, and KQ designed the shielding device and analysis data. QunZ, XX, YW, KY, NW, and HW designed the study and reviewed the manuscript. LiL and JW prepared manuscript. All authors contributed to the article and approved the submitted version.

## Funding

This study was supported by the National Key R&D Program of China (2021ZD0202805 and 2019YFA0709504), the Natural Science Foundation of China (NSFC, nos. 81972141, 81972140, 82002392, 82172544 and 31900719), Shanghai Municipal Key Clinical Specialty (nos. SHSLCZDZK02702), and Shanghai Sailing Program (20YF1403500), the Innovative Research Team of High-level Local Universities in Shanghai, 111 Project (B18015), Shanghai Municipal Science and Technology Major Project (2018SHZDZX01), and Shanghai Center for Brain Science and Brain-Inspired Technology.

## Conflict of interest

SG, QuanZ, and KQ were employed by Nanjing Vishee Medical Technology Co., Ltd.

The remaining authors declare that the research was conducted in the absence of any commercial or financial relationships that could be construed as a potential conflict of interest.

## Publisher’s note

All claims expressed in this article are solely those of the authors and do not necessarily represent those of their affiliated organizations, or those of the publisher, the editors and the reviewers. Any product that may be evaluated in this article, or claim that may be made by its manufacturer, is not guaranteed or endorsed by the publisher.
